# Development and Validation of Conditions for Extracting Flavonoids Content and Evaluation of Antioxidant and Cytoprotective Activities from *Bougainvillea* x *buttiana* Bracteas (var. Rose)

**DOI:** 10.3390/antiox8080264

**Published:** 2019-08-01

**Authors:** Rodolfo Abarca-Vargas, Alejandro Zamilpa, Vera L. Petricevich

**Affiliations:** 1 Facultad de Medicina de la, Universidad Autónoma del Estado de Morelos (UAEM), Calle: Leñeros, esquina Iztaccíhuatl s/n. Col. Volcanes, Cuernavaca, Mor. C.P. 62350, Mexico; 2 Centro de Investigación Biomédica del Sur, Instituto Mexicano del Seguro Social (IMSS), Argentina No. 1, Xochitepec CP 62790, Morelos, Mexico

**Keywords:** rutin, quercetin-O-glucoside, quercetin-rhamnoside, extraction, *Bougainvillea* x *buttiana*, antioxidant activity, cytoprotective activity

## Abstract

In this study the effect of the ethanol concentration of *Bougainvillea *x* buttiana* extracts on the flavonoids content, and its antioxidant and cytoprotective activities in vitro were determined and compared. For the elucidation of the chemical constituents, the high-performance liquid chromatography method (HPLC) was used, and verification of the antioxidant activity was carried out using the 2,2-diphenyl-1-picrylhydrazyl (DPPH) free radical method. The cytoprotective effects of extracts were determined by exposure to hydrogen peroxide. The HPLC analysis showed the presence of rutin, quercetin-3-glucoside and quercetin rhamnoside. Among the extracts investigated the best recuperation of the rutin content was observed in extracts with 80% ethanol (83 ± 5 mg/mL). The amounts of rutin present in all extracts contribute to the antioxidant capacity and the IC_50_ was 427.49 (0%), 275.41 (50%), 271.61 (80%), and 272.14 (100%) µg/mL. The lowest percentage of viability was found in the cultures exposed to 100% ethanol (92%). In cultures exposed to hydrogen peroxide the percentages of protection were 25%, 33%, 78%, and 65% for cultures treated for 72 h at 0%, 50%, 80%, and 100% ethanol, respectively. The ethanolic extract of *B.* x *buttiana* was confirmed to have high rutin content with potent antioxidant activity, low cytotoxic and strong cytoprotective effects.

## 1. Introduction

*Bougainvillea* x *buttiana* (Bxb) is cultivated for ornamental and medicinal purposes in Mexico. Phytopharmacological studies have shown anti-inflammatory, anti-nocipetive, immunostimulant, and antioxidants activities [1,2,3]. The chemical compounds of *B*. x *buttiana* encompass a few compounds [3]. The organic substances present in the plants extracts exhibited natural variations in composition because of factors such as season, geography and cultivar that can have a substantial effect on the quality and the restoration fee of herbal drug treatments [4]. Although Bxb is inserted in medicinal plants of Mexico, its extract has not been carried out studies for the analytical validation of its chemical constituency. Separation techniques such as high-performance liquid chromatography (HPLC) are used for validation through the ability to identify compounds in a comparison with standards of known purified substances. Therefore, it is far very important to provide quality control parameters to insure their efficacy and protection. In recent years, loads of biochemical and pharmacological properties of plants extracts have been associated to flavonoid content [5].

Flavonoids represent a group of naturally diverse polyphenols involved in the chemical defense of plants against various predators. They are widely known and studied because of their innumerable biological activities that confer beneficial effects on human health [6]. Several studies have shown the existence of more than 4000 types of flavonoids, being divided into flavonoids, flavones, catechins, flavanones, anthocyanins, and chalcones [7]. Among the numerous functions of flavonoids is the antioxidant activity, which is due to its sequestering action of free radicals and by chelating metal ions, in this way are able to protect the tissues of free radicals and lipid peroxidation [5,6,7]. Several structural forms are found in flavonoids, but their basic structure consists of fifteen carbon atoms in their nucleus, arranged in three rings (C6-C3-C6), two substituted phenol rings (A and B), and one pyran (heterocyclic C chain) coupled to ring A.

In this work we emphasize rutin, quercetin glucoside, and quercetin rhamnoside which is a flavonoid that has a significant antioxidant activity with important tissue protection capacity [8]. Chemically its name is quercetin-3-rutinoside, and it is sparingly soluble in water but freely soluble in methanol. Quercetin-3-rutinoside has been proven to have a spread of pharmacological properties useful health effects including anticarcinogenic, antifungal, anti-inflammatory, anti-mutagenic, anti-tumor, antiviral, partial defensive effect on opposition the diabetes development, hepatoprotective, vaso-protective, and analgesic [9,10,11,12,13,14,15,16]. Different uses have been attributed to flavonoids in especial rutin, as an oxidation inhibitor, a natural dyeing agent, in cosmetology as a protector against sunburn and in food applications [12,13,14,15,16]. There is published information of specific components of the qualitative composition of flavonoids and their contents in incipient flower, but up to date there has been scarce information available concerning the variations in flavonoids content in the different plant parts or populations [17,18,19].

Many of the biological actions of flavonoids may be related to their reducing properties and their chemical structure, which neutralizes or sequesters free radicals, chelating the transition metals and acting both at the beginning and in the transmission of the oxidation process [20,21].

The exact mechanisms by which flavonoids exert their useful properties, often antioxidants, need to be widely recognized because currently available information is insufficient and the main antioxidant assays used are carried out in vitro. In the scientific literature, there are many studies related to the in vitro antioxidant activity of flavonoids, and this information serves as the basis for in vivo studies [12,17,18,19,20]. The antioxidant evaluation of the cell survival through living cells and physiologically relevant oxidants are of crucial importance [12]. Considering the role of free radicals in the development of pathological approaches of numerous illness as well as the importance of the use of efficient antioxidants for preventive and therapeutic purposes [12].

This work aimed at the chemical isolation of flavonoids and the development of an analytical methodology to determine and quantify these compounds in distinctive ethanol extracts of the *Bougainvillea* x *buttiana* bracts. For optimization of flavonoids in different ethanolic extracts from *B*. x *buttiana* the HPLC technique was evaluated and demonstrated. In addition, the impact of these extractions at the yield of antioxidant and cytoprotector activities was studied.

## 2. Materials and Methods

### 2.1. Chemicals Reagents

Absolute ethanol (>99% *v/v*) was obtained from Golden Bell (Zapopan, Jalisco, Mexico). Tween-20, gallic acid, dimethyl sulfoxide (DMSO), quercetin, rutin, Ciocalteu’s Folin Phenol Reagent, sodium nitrite, 3-(4,5-dimethylthiazol-2-yl)-2,5-diphenyltetrazolium bromide (MTT), sodium carbonate and Iron (III) chloride hexahydrate were purchased from Sigma Aldrich (Toluca, México). All experiments were performed with chemical reagents of analytical grade, and the deionized water was obtained from a Milli-Q water purification system (Millipore Corporation, Bedford, MA, USA).

### 2.2. Preparation of Bougainvillea x buttiana Extract

The aerial parts (flowers) used in this study were collected from Temixco, Morelos (18°52′20.1″ N and 99°14′40.6″ W, 1185 m), and classified as *B*. x *buttiana* (var. Rose) and retained at the Herbarium HUMO, CIByC (UAEM) as a voucher specimen (33,872). *B*. x *buttiana* (Var. Rose) bracts (BxbR) were collected, dehydrated, and evaluated in accordance with the method detailed described by Abarca-Vargas et al. in 2016 [12]. Briefly, 500 g of bracts from BxbR were collected, air-dehydrated at 25 °C per 7 days and promptly exposed to an electric grinder to produce first-quality powder. The different concentrations of ethanol were adjusted and after extraction, each obtained extract was filtered using a Whatman n° 1 filter (Whatman International, Buckinghamshire, UK). All extraction runs were executed in triplicate.

### 2.3. Determination of Flavonoids

#### 2.3.1. Samples Preparation

One milligram of the ethanol extract of each concentration was dissolved in 1 mL HPLC grade methanol and then filtered using a membrane filter.

#### 2.3.2. Standard Solution Preparation

A stock solution was prepared by 1 mg of rutin in 1 mL HPLC grade methanol. Aliquots of the stock solution (1 mg/mL) were then diluted in a volumetric flask, each separately with methanol up to 1 mL.

#### 2.3.3. Determination of Flavonoids by High Performance Liquid Chromatography (HPLC)

Chromatographic evaluation was executed on a Waters 2695 Separation module system equipped with a Waters 996 photodiode systematized detector and Empower Pro software (Waters Corporation, Milford, MA, USA). Chemical segregation was achieved usage a Supelcosil LC-F column with the following specifications 4.6 mm × 250 mm i.d., 5 µm particle size (Sigma Aldrich, Bellefonte, PA, USA). The mobile phase be expressed by of 0.5% trifluoroacetic acid aqueous solution (solvent A) and acetonitrile (solvent B). The gradient system was as follows: 0–1 min, 0% B; 2–3 min, 5% B, 4–20 min, 30% B; 21–23 min, 50% B 14–15 min; 24–25 min, 80% B; 26–27 min, 100% B; and 28–30 min, 0% B. The flow rate was maintained at 0.9 mL/min, and the sample injection volume was 10 µL. Flavonoids were measure at 350 nm. Four ascendant concentration (25, 50, 100 and 200 µg/mL) of a commercial standard of rutin and quercetin (Sigma-Aldrich) were injected by triplicate in the chromatographic method to build the calibration curve (*Y* = 8646.3*X* + 96813, *R*^2^ = 0.9984). The linearity plot was also evaluated, to verify the values obtained for quantification limit. An analytical curve was constructed from the rutin standard solution concentrations which were close to the expected quantification limits.

#### 2.3.4. Calibration Curve

The curve was constructed by injecting, in triplicates, four concentrations of stock solution (25, 50, 100, and 200 mg/mL). The regression equation and the coefficient of the correlation (*Y*= 8646.3*X* + 96813; *R*^2^ = 0.9983) were then derived from the curve to ensure that it accurately describes the relationship between the estimated response (*Y*) and the standard concentration (*X*). The linearity plot was also evaluated, to verify the values obtained for quantification limit. An analytical curve was constructed from the rutin standard solution concentrations, which were close to the expected quantification limit.

### 2.4. Estimation of Total Flavonoid Content (TFC)

Total flavonoids contents found in BxbR extracts with diverse concentrations of ethanol have been obtained as technique described by Zhishen et al., 1999 [22]. The assay consisted by means of 0.5 mL of extract that was mixed with 75 μL of 5% NaNO_2_ solution and incubated for 6 min, at 25 °C. The solution constituted by 150 μL of a 10% AlCl_3_·6H_2_O was added and incubated for another 5 min. In sequence, 0.5 mL of 1 M NaOH and 2.5 mL of distilled water was combined. All solutions were blended, and their absorbance changed into obtained at 510 nm using spectrophotometer. TFC expressed as EQmg/g of extract by performing from three different assay. Based on the calibration curve the following equation was obtained: (*Y* = 2.2224*X* + 0.982, *R*^2^ = 0.9917) in which *X* and *Y* become the absorbance and the EQmg/g, respectively.

#### 2.4.1. Experimental Schedule

The optimization of rutin present in BxbR extracts was studied by analyzing the effects of ethanol concentration. The crude extract was analyzed for antioxidant activity using the DPPH radical scavenging assay.

#### 2.4.2. In Vitro Antioxidant Activity by DPPH Method

The 2,2-diphenyl-1-picrylhydrazyl (DPPH)-free radical activity of the extracts with different amounts of ethanol 0%, 50%, 80%, and 100% was performed using the method previously described by Miliauskas et al., 2004 [23]. Briefly, each extract was prepared from serial quantities of 7.3–500 µg/mL and mixed with equal volumes of methanolic DPPH (0.1 mM). The standard curve in the range of 37.5–150 µg/mL of quercetin was used. All samples were maintained in the dark at 25 °C for 30 min. Absorbances of the control and each extract were performed in triplicate and compared to methanol containing DPPH (as blank) at 517 nm using a UV light spectrophotometer. Percent inhibition of DPPH radical scavenging capacity was calculated by using of equation: DPPH scavenging % = [(Abs_sample_ – Abs_blank_/Abs_control_) × 100]. All results were expressed in µM quercetin equivalent antioxidant capacity per 100 g dry weight sample (µMQE/gram of dried extract). The calibration equation for quercetin was *Y* = −5.6441*X* + 1.7778, *R*^2^ = 0.9998. The IC_50_ indicates the quantity of antioxidant capable of lowering the DPPH concentration by 50% which was calculated by liner regression equation between the extract concentration and the corresponding scavenging effect. Thus, a lower IC_50_ values indicate higher antioxidant activity.

#### 2.4.3. Antioxidant Activity Index (AAI)

The antioxidant activity index (AAI) was estimated through the use of the following formula: AAI = DPPH final concentration of DPPH in the control sample µg/mL/IC_50_ µg/mL. As follows, the values of AAI were estimated in consideration of the DPPH mass and the compound tested mass in the reaction derive in a constant for each compound independent of the concentration of DPPH and pattern use. In accordance with the method established by Scherer and Godoy, 2009 [24], the AAI values for plants extracts were considered as poor activity <0.05, moderate activity <1.0, strong activity <2.0, and very strong activity >2.0.

### 2.5. Cell Proliferation and Viability Assay Using L929 Fibroblast

L929 mouse fibroblast cells NCTC clone L929, [L-929, derivate of strain L] (ATCC CCL-1, Manassa, VA, USA) were seeded at a density of 1–5 × 10^3^ cells/well into a 24-well plate in RPMI-1640 culture medium supplemented with 10% FBS and incubated at 37 °C and 5% CO_2_. After 24 h of incubation, the culture medium was changed into replaced by using fresh RPMI-1640 with 10% FBS. The cells were exposed to a different concentration at 0, 5, 10, 25, 50, 100, and 200 µg/mL (dry weight) of each extract. For culture control the culture medium was changed and replaced by fresh RPMI-1640 with 10% FBS. Cells were incubated for 24, 48, and 72 h, at 37 °C and 5% CO_2_. Subsequently, the culture medium was discarded and replaced by 100 µL of fresh RPMI-1640 with 10 µL of 3-(4,5-dimethylthiazol-2-yl)-2,5-diphenyltetrazolium bromide (MTT) solution (5 mg/mL in PBS) per well and incubated in the dark, for 3 h, at 37 °C, and 5% CO_2_. A negative control without cells with 100 µL of RPMI-1640 and 10 µL of MTT solution was performed. Subsequently, 85 µL of culture medium was removed and 50 µL of DMSO was added onto each well and incubated for more 10 min, at 37 °C, in a humidified 5% CO_2_ atmosphere. After homogenizing formazan crystals, the absorbance at 540 nm was determined by an ELISA plate reader. The percentage of cell proliferation/viability was calculated and compared to control (100% of viability).

#### Hydrogen Peroxide-Induced Oxidative Stress in L929

Hydrogen peroxide was used for induction of oxidative stress as described by Balekar et al. [25] and Ponnusamy et al. [26]. The L929 fibroblast cells were seeded at a density of 1–5 × 10^3^ cells/well into a 24-well plate in RPMI-1640 supplemented with 10% FBS and incubated for 18 h at 37 °C, in a humidified 5% CO_2_. A curve with 5 H_2_O_2_ concentrations such as (0.0625, 0.125, 0.25, 0.5, and 1.0 mM) was performed to determine the H_2_O_2_ amount which decreases cell viability by 80% after 24 h of exposure using MTT assay. In the experimental conditions the concentration capable to decrease 80% of cellular viability was 1.0 mM of H_2_O_2_. Subsequently, L929 fibroblast cells were seeded at a density of 1–5 × 10^3^ cells/well into a 24-well plate containing RPMI-1640 medium supplemented with 10% FBS and incubated for 18 h at 37 °C, in a humidified 5% CO_2_. After this period of incubation, RPMI-1640 medium with 10% FBS containing 1 IC_50_ values µg/mL (dry weight) of each correspondent extract was used to treat cells in different times as described in Table 1.

### 2.6. Statistics

Statistical analysis was calculated in triplicate analysis, and the all results were determined as the mean standard deviation of three experiments, each experiment was performed in triplicate for each treatment. Statistically significant variations among extracts and samples were calculated using STATISTICA (STATSOFT, 2004, Statsoft, Tulsa, OK, USA, and variance was evaluated using ANOVA and the Tukey test at the level of 5% (*n* = 3; *p* < 0.05). The correlations among information acquired from the phenolic composition and antioxidant activity were determined using the Pearson correlation coefficient (*r*).

## 3. Results

### 3.1. Phytocompound Analyses

In previous publications we have shown by GC-MS the phytocompounds present in the extracts of *B.* x *buttiana* Abarca-Vargas et al., 2016 [3] (Table 2).

### 3.2. Total Flavonoids Contents

**Figure 1:**
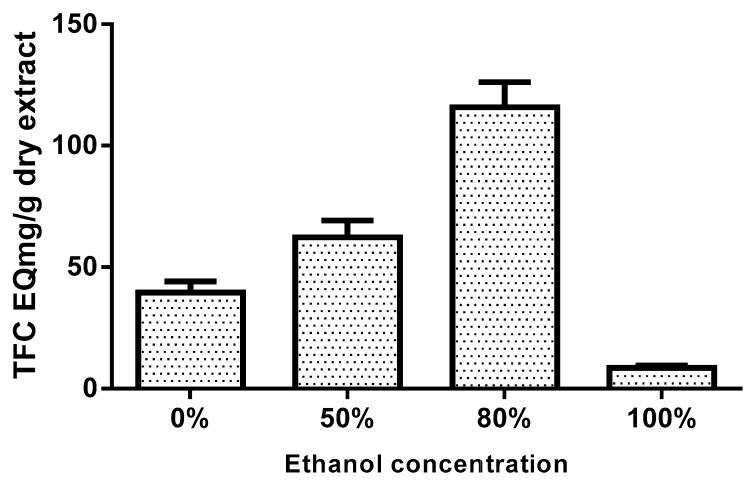
Comparison of total flavonoids content in ethanolic extracts. Samples of *Bougainvillea* x *buttiana* extract ethanol 0%, 50%, 80%, and 100% at 26 °C for 24 h were assayed to determine the concentration of total phenolic content as described in Materials and Methods. Each bar corresponds to the results (mean ± standard deviation (SD)) from three different preparations.

The flavonoids concentration of flavonoids in BxbR extracts with different ethanol amounts was tested by spectrophotometric technique with aluminum chloride, and expressed in terms of mgQE/g of extract (Figure 1). The flavonoids concentration in extracts with different concentrations of ethanol ranged from 39.5 to 115.8 mg/g. The concentration of flavonoids observed for the extracts with 0%, 50%, 80%, and 100% EtOH was 39.5, 62.3, 115.8, and 78.4 mgQE/g, respectively.

### 3.3. Determination of Flavonoids by HPLC

**Figure 2:**
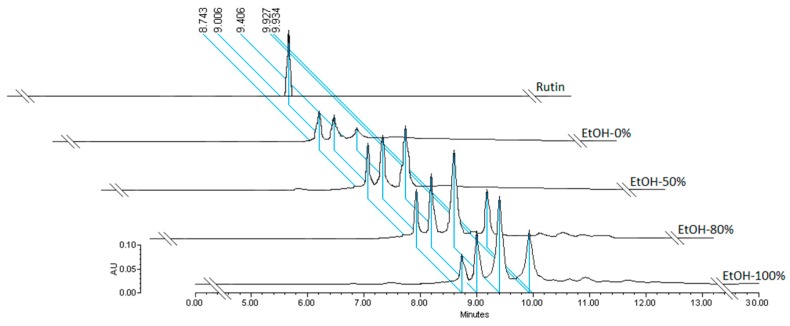
HPLC of Rutin. Chromatographic profile of the routine concentration curve (standard) and extracts of EtOH-0%, EtOH-50%, EtOH-80%, and EtOH-100%.

Through the analysis of the chromatograms, it was possible in all extracts with EtOH 0%, 50%, 80%, and 100% to confirm the presence of three flavonoids including rutin, quercetin glucoside, and quercetin rhamnoside as identified by HPLC-UV. Quantification of rutin, quercetin glucoside and quercetin rhamnoside in extracts with 0%, 50%, 80%, and 100% EtOH was done by HPLC method as shown in Figure 2. The rutin peak retention time was found to be the same while being injected several times, giving a symmetric and well-resolved peak. The runtime was 30 min for the whole chromatogram, the retention time of rutin was 9.006 min, and rutin appeared on the chromatograms of extracts at 0%, 50%, 80%, and 100% EtOH. In the same extracts, the presence of quercetin glucoside and quercetin rhamnoside was confirmed at 9.4 and 10.1 min, respectively. This indicates that the developed method is convenient and rapid.

**Figure 3:**
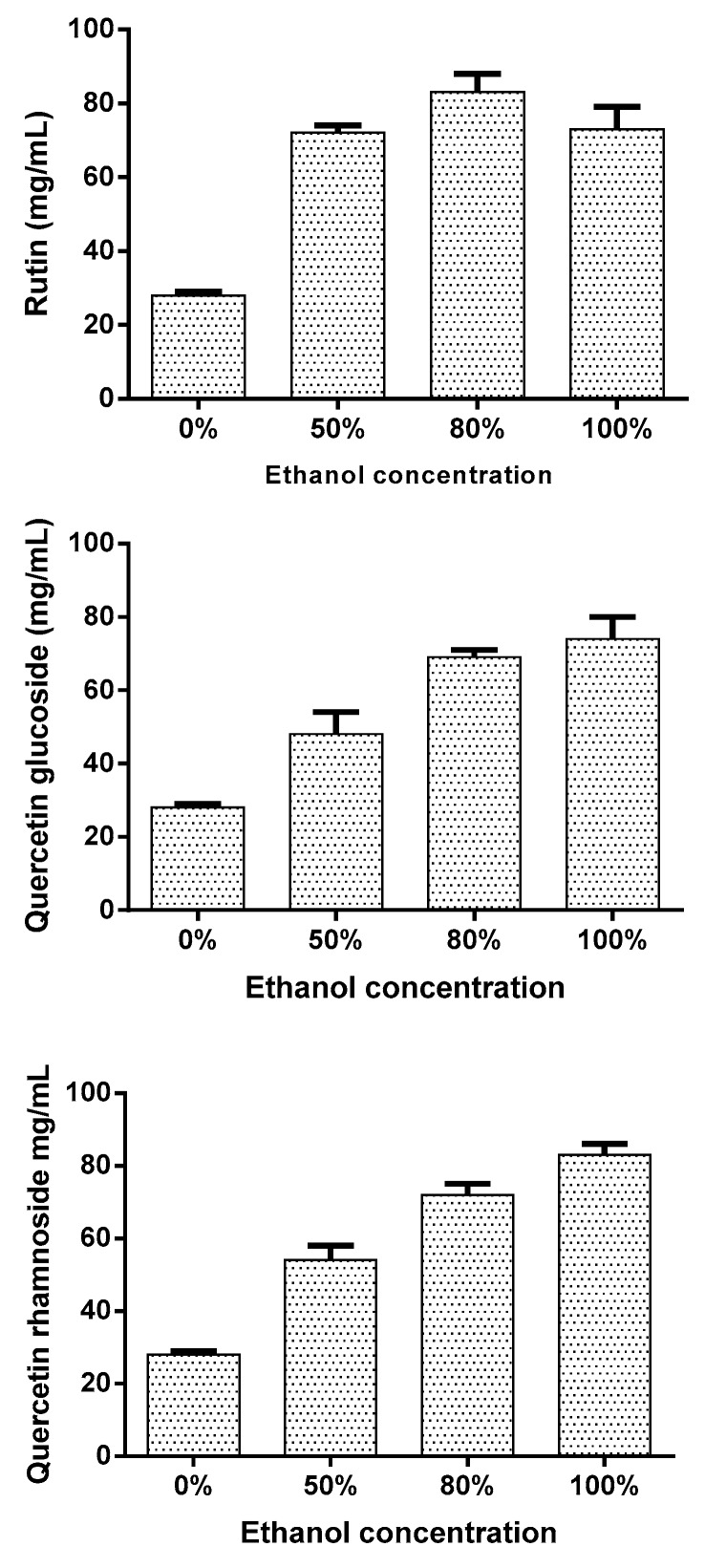
Flavonoid determination. The yield of rutin, quercetin glucoside and quercetin rhamnoside in different ethanol extracts was determine. Individual samples of *B.* x *buttiana* extract ethanol 0%, 50%, 80%, and 100% were investigated to determine the yield of rutin as described in Materials and Methods. Each bar corresponds to the results (mean ± standard deviation (SD)) from three different preparations.

There were important variations among different extracts concerning their rutin, quercetin glucoside and quercetin rhamnoside contents. The extracts with 0%, 50%, 80%, and 100% EtOH were investigated for their contents of rutin as shown in Figure 3. Rutin contents were significantly higher in extracts with 50%, 80%, and 100% EtOH when compared to extracts prepared with 0% (*p* < 0.001). The highest yield of rutin was obtained in the extract with 80% EtOH. With respect to quercetin glucoside and quercetin rhamnoside, the highest concentrations were obtained in extracts with 100% EtOH.

### 3.4. Determination Antioxidant Activity

**Figure 4:**
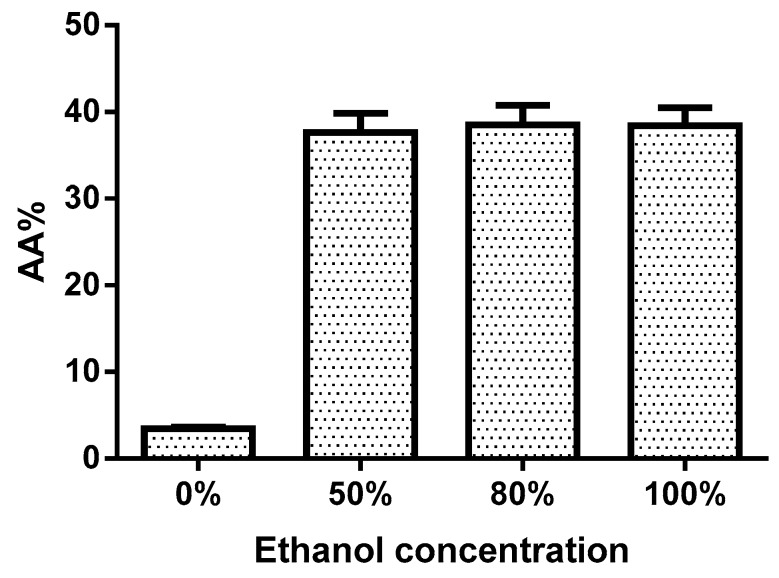
Antioxidant activity percentage. Individual samples of *B*. x *buttiana* extract ethanol 0%, 50%, 80%, and 100% were investigated to determine the antioxidant activity as described in Materials and Methods. Quercetin was used as reference antioxidants. Each bar corresponds to the results (mean ± standard deviation (SD)) from three different preparations.

To determine the antioxidant activity, the extracts prepared with different concentrations of ethanol were evaluated by the DPPH method assay, and quercetin was used as reference. The quercetin standard amount of 100 µg/mL caused an inhibition percentage of 50% (data not shown). The extracts with 0%, 50%, 80%, and 100% EtOH exhibited the percentages of antioxidant activity 3.49%, 37.66%, 38.52%, and 38.40%, respectively. The percentage of antioxidant activity in the extracts prepared with different amounts of ethanol were significantly higher when compared to extracts prepared without ethanol. The results presented in Figure 4 reveal that the extracts were competent of diminishing the radicals to the corresponding hydrazine when it reacts with the hydrogen donors.

### 3.5. IC_50_ Value of DPPH Radical Scavenging Activity and Activity Antioxidant Index

**Figure 5:**
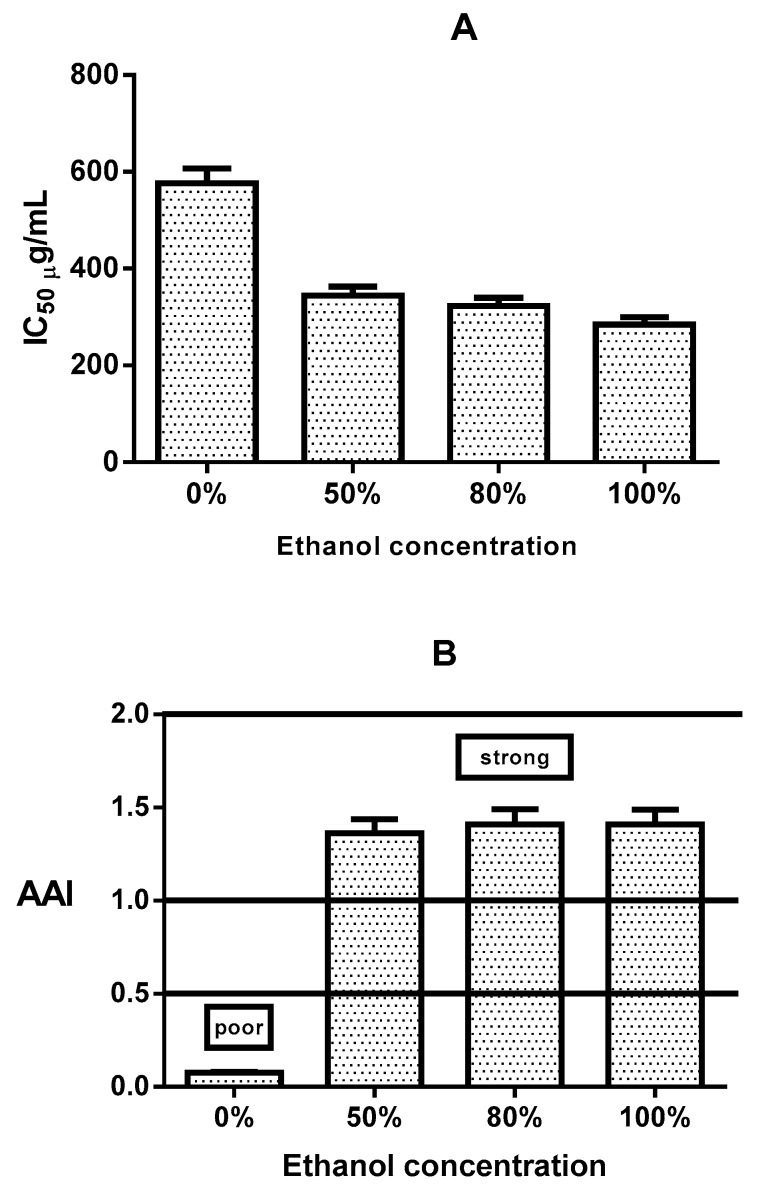
IC_50_ values and antioxidant activity index (AAI). Individual samples of *B*. x *buttiana* extract ethanol 0%, 50%, 80%, and 100% were investigated to evaluate the IC_50_ (**A**) and antioxidant activity index (**B**) as described in Materials and Methods. Each bar corresponds to the results (mean ± standard deviation (SD)) from three different preparations.

The values of IC_50_ were calculated to evaluate the extract concentration required to inhibit 50% of the radical. A decrease in the IC_50_ value corresponds to higher the antioxidant activity of the extracts. In Figure 5 are shown the values of IC_50_ obtained from extracts of *B.* x *buttiana* prepared with distinct concentrations of ethanol. The obtained IC_50_ values were 0% EtOH (427.49 µg/mL), 50% EtOH (275.41 µg/mL), 80% EtOH (271.61 µg/mL), and 100% EtOH (272.14 µg/mL). The extracts prepared with different amounts of ethanol IC_50_ values were significantly lower when compared to those extracts without ethanol (*p* < 0.001). The IC_50_ values obtained in the extracts prepared with 50%, 80% and 100% EtOH were similar (Figure 5A).

The antioxidant activity index values are described in Figure 5B. All extracts of *B.* x *buttiana* showed antioxidant activity at different levels. The extracts prepared 0% EtOH showed poor antioxidant activity. In contrast, extracts prepared with different amounts of ethanol had strong antioxidant activities.

### 3.6. Ethanol Concentration Effect on the Cellular Viability

**Figure 6:**
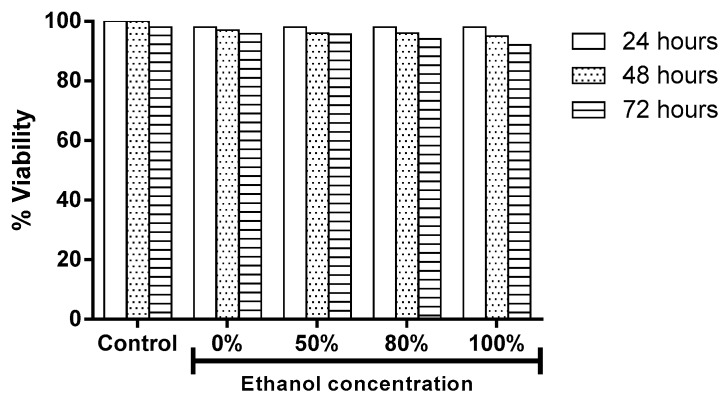
Ethanol concentration effect on the cellular viability. Individual samples of *B*. x *buttiana* extract with 1 mg/mL of each extract 0%, 50%, 80%, and 100% for 24, 48, and 72 h were investigated to determine the viability percentage by MTT test as described in Materials and Methods. Each bar corresponds to the results (mean ± standard deviation (SD)) from three different preparations.

The effect of the extracts on the viability of cells L929 was measured using the MTT method after 24, 48, and 72 h of incubation with extract concentrations of 1 mg/mL. The viability percentages in cell cultures treated with the extracts for 24 and 48 h were 98% and 96%, respectively. The effects of extracts on cell cytotoxicity at different concentrations of ethanol treated for 72 h are shown in Figure 6. The percentage of viability decreased in a manner dependent on increasing ethanol concentration in the three cultures studied here (Figure 6).

### 3.7. Ethanol Concentration Effect on H_2_O_2_ Induced Oxidative Stress and Cell Survival

**Figure 7:**
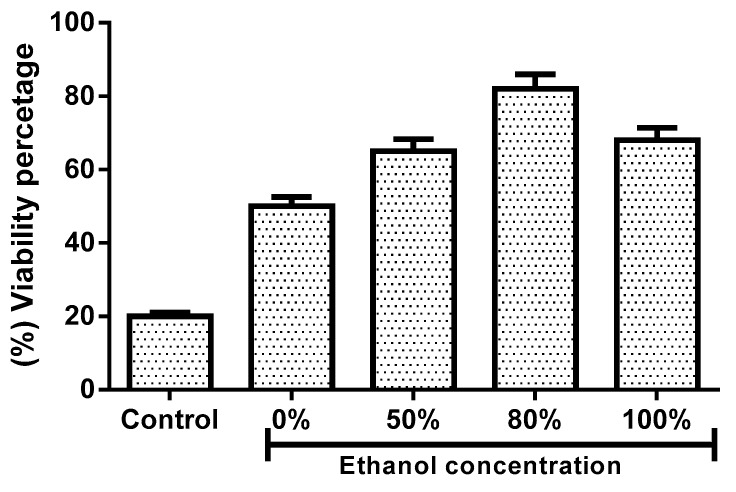
Extract ethanol concentration effect on cell survival. Groups of cells were treated with 1 mM of H_2_O_2_ to induce oxidative stress for 24 h at 37 °C with 5% CO_2_. After this incubation, the cells were treated with 1 IC_50_ individual extract ethanol of 0% (427.49 µg/mL), 50% (275.41 µg/mL), 80% (271.61 µg/mL), and 100% (272.14 µg/mL) and incubated for 24 h to determine the viability percentage as described in Materials and Methods. Each bar corresponds to the results (mean ± standard deviation (SD)) from three different preparations.

The effect of the different concentrations of extracts 0%, 50% EtOH, 80% EtOH, and 100% EtOH on cell survival pretreated with H_2_O_2_ is shown in Figure 7. In cells treated for 24 h with different concentrations of ethanol extracts, cell survival was significantly higher when compared with those cells nontreated (*p* < 0.01). The highest cell survival (82%) was found in groups of cells treated with 80% EtOH extract.

**Figure 8:**
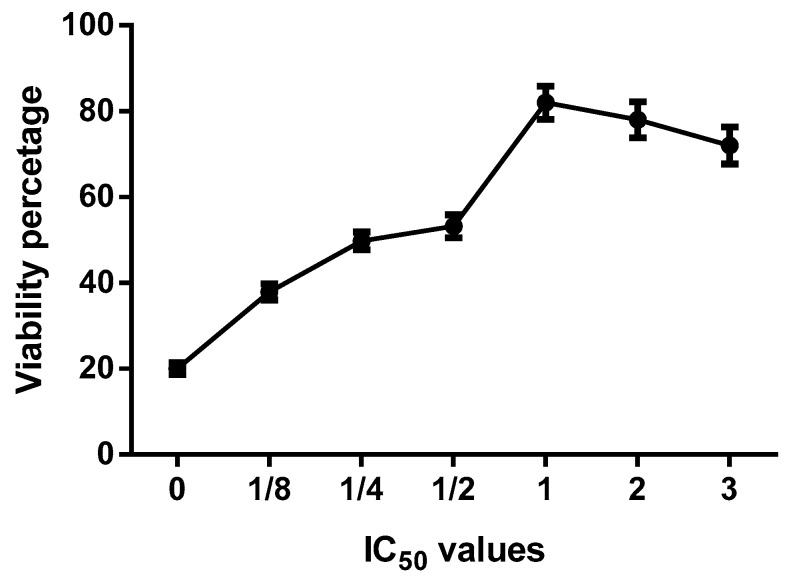
Extract 80% EtOH effect on cell survival. Groups of cells were treated with 1 mM of H_2_O_2_ to induce oxidative stress for 24 h at 37 ºC with 5% CO_2_. After this incubation, the cells were treated with extract 80% EtOH at different IC_50_ values were assayed to determine the viability percentage as described in Materials and Methods. Each point corresponds to the results (mean ± standard deviation (SD)) from three different preparations.

When comparing all extracts, we found that the extracts prepared with 80% EtOH showed a greater amount of rutin, a slight decrease in IC_50_ values, a similar index of antioxidant activity and high cellular viability (Figure 8). To determine the percentage of cytoprotection, L929 cell cultures treated with H_2_O_2_ were exposed to different IC50 values of the extracts prepared with 80% EtOH for 24 h. 8 shows that, in the presence of the extract, the percentage of cell viability increases, which differs significantly from that of the control. The highest increase in the viability percentage was obtained at a concentration equivalent to 1 IC_50_ corresponding to 271.61 μg/mL. With the increase of IC_50_ values, a discrete decrease in the viability percentage was observed (Figure 8).

## 4. Discussion

*Bougainvillea* x *buttiana* has been reported to contain different compounds, such as alkanes, esters, flavonoids, fatty acids, phenolic, and terpenes, that are well known for their antioxidant and anti-inflammatory properties, which are believed to play an important role in many diseases Traditionally, *Bougainvillea* has been used for the treatment of different diseases [1,2,3]. Different extraction methods are used to obtain crude extracts from plant materials. However, there is no universal extraction protocol, one possible explanation would be through the specific requirements of each plant for optimal recovery of compounds, especially those responsible for biological activities. Previously, we have shown by GC-MS the presence of 15 compounds such as phenolics, alkanes, esters, terpenes, and fatty acids [3]. The extraction efficiency and pharmacological activities are dependent on the efficiency of solvent polarity due to the presence of various compounds with varied chemical characteristics [27,28,29]. Several solvents are used for the extraction of compounds with therapeutic potential, as in the case of pure ethanol or combined with water [30]. In this study, we evaluated the effect of ethanol concentration on the yield of total flavonoids. The total flavonoids content in different BxbR extracts with different ethanol amounts was assayed using spectrophotometric method with aluminum chloride. The highest TFC was observed in the extracts prepared with 80% EtOH with 115.8 mgQE/g. Several non-phenolic compounds can also be extracted and contribute to this yield, possibly due to the high aqueous solubility of proteins and carbohydrates [30]. The mixture of organic solvent and water can promote the extraction of chemicals that are more soluble in water and/or organic solvent. Our results suggest that, below a certain limit, the extraction efficiency can be improved by increasing the diffusion rate and solvent solubility [31,32,33]. Studies on the chemical composition of vegetal species of medicinal interest, with the purpose of identifying the groups of relevant secondary metabolites, contribute to the elucidation of the mechanisms that aim to prove these activities.

In this work for identification and characterization of substances presents in extracts from *B.* x *buttiana* can be facilitated by using HLPC. The validation method of quantitative HPLC analysis of flavonoids is shown in Figure 2. The significant rate of recovery of flavonoids was characterized by precision, accuracy, and meticulousness, and can be used for the qualitative and quantitative assay of flavonoids present in the different extracts from *B*. x *buttiana*. The identification and quantification of different flavonoids present in extracts with different concentrations of ethanol were shown in Figure 2; Figure 3. The HPLC chromatogram of the *B*. x *buttiana* ethanol extract showed the presence of rutin, quercetin glucoside, and quercetin rhamnoside. Among the extracts with 50%, 80%, and 100% EtOH analyzed an important amount of rutin was observed. Slightly higher values were obtained in the extracts prepared with 80% EtOH. The solvent could be hydrolize the glucoside and restore the quercetin form. These findings were similar results have previously been reported, indicating the efficiency of ethanol in the extraction of rutin [34]. The highest concentrations of quercetin glucoside and quercetin rhamnoside were obtained in the extracts with 100% EtOH, indicating that the polarity reduction favors the yields of these flavonoids.

Flavonoids are known for their anti-inflammatory, vasoactive properties, inhibition of low-density lipoprotein peroxidation, and also is considered a potent total antioxidant, reducing power and chelating activity [35,36]. Antioxidants are referred to as substances that prevent oxidation and may be endogenous and exogenous in origin. Flavonoids with antioxidant activity are dependent on the chemical structure of the compound [37,38]. The total flavonoid content in the extracts from BxbR bracts may be associated with antioxidant activity.

Flavonoids such as rutin, quercetin glucoside and quercetin rhamnoside are used for the prevention and cure of various diseases, which is mainly associated with free radicals. In this study, the extracts of *B*. x *buttiana* prepared with 0%, 50%, 80%, and 100% EtOH showed antioxidant activity with a percentage of 3.49%, 37.66%, 38.52%, and 38.40%, respectively. The IC_50_ values were similar among the extracts prepared with 50%, 80%, and 100% EtOH and significantly lower when compared to the values found in the extracts with 0% EtOH. AAI by the DPPH method was used because it is considered appropriate for comparing extracts. In extracts prepared with 50%, 80%, and 100% EtOH no difference in AAI values was observed. These extracts the presented strong AAI (between 1 and 2) according to the criteria established by Scherer and Godoy in 2009 [24] for plant extracts.

Under standard physiological conditions, mobile oxidative damage is prevented by way of antioxidant defenses that neutralize reactive oxygen species. Under oxidative stress conditions, the cellular inability to deal properly with the reactive oxygen species is verified, which causes the cell to suffer damage. For this reason, there is a great interest in the use of natural antioxidants as potential therapeutic agents capable of neutralizing the reactive species and consequently oxidative damages. The in vitro characterization of oxidizing compounds provides an indication of the ability of a substance to remove free radicals and does not indicate the effect of an antioxidant on cell survival.

Some in vitro studies have evaluated the cytotoxic effects of the plant extracts by means of cell cultures. Cytotoxic assays in cell cultures can help evaluate the effects resulting from a specific concentration of an agent through the damage caused to either cell structure or biochemical pathways within the cells [39]. In the present study the effect of the extracts on viability percentage was studied in L929 cells by MTT method. Under experimental conditions, the percentage of viability decreased in a manner dependent on the increase in ethanol concentration at 72 h.

Hydrogen peroxide-induced oxidative stress is an alternative to evaluate the antioxidant activity of extracts in cells. In this study, we used L929 cells to evaluate the antioxidant capacity of extracts of *B*. x *buttiana* prepared with different amounts of ethanol at the cellular level, and the results obtained show that the different extracts significantly decrease the oxidative damages in these cells, suggesting that the presence of compounds including, the rutin, quercetin glucoside, and quercetin rhamnoside have a high antioxidant capacity in vivo. These results suggest that high levels of hydrogen peroxide could be reduced with the presence of these extracts. In presence of 0% EtOH, which has the lowest amount of rutin, quercetin glucoside, and quercetin rhamnoside, the L929 cells were deficient in antioxidant defenses, presenting growth inhibition. The extracts prepared with 80% EtOH demonstrated better protection against damage caused by hydrogen peroxide. These results are in agreement with those obtained in keratinocytes and fibroblast [40]. These authors assigned this effect to the antioxidant activity of anthocyanins, metabolites that belong to phenolic group and are found in abundance in different fruits. A compound can exert its antioxidant actions in vivo in a number of ways: through inhibition of the production of reactive species or the direct elimination of reactive species [41]. The antioxidant capacity of extracts prepared with 80% ethanol in the treatment of cells improved the activity by up to 82% when compared to the results of hydrogen peroxide, contributing at least in part to the levels of resistance observed in the induction of the response to the oxidative stress.

## 5. Conclusions

The HPLC method used was simple, precise, sensitive and accurate. This method was successfully applied for the routine evaluation in 5 different extracts of *B*. x *buttiana* that can be used for the analysis of quality control. The optimal extraction condition to achieve the best results in terms of antioxidant activity yield, total flavonoids and rutin, quercetin glucoside, and quercetin rhamnoside content is 80% EtOH as extraction solvent, taking into consideration the balance between the production of reactive oxygen species and their detoxification and the fact that they are fundamental for the vitality of biological systems. When this equilibrium is disturbed, it has the capacity to increase the production of peroxides and free radicals that cause damage to cellular components. This study shows, through the index of antioxidant activity, that the extracts prepared with 50%, 80%, and 100% EtOH showed a strong antioxidant activity. The in vivo antioxidant activity results demonstrate the importance of these extracts in the protection of the cell line L929 when under oxidative stress. Our results show that the extracts 50%, 80%, and 100% EtOH significantly reduced oxidative damage, which was important in the protecting against induced oxidative stress. The highest protection against oxidative stress was observed in the cell cultures treated with the extract 80% EtOH. Through the obtained results, we conclude that extraction with 80% ethanol can be a therapeutic alternative for the recognized antioxidant activity and with low toxic potential.

## Figures and Tables

**Table 1:** Treatment for oxidative stress determination.

Experiments	Treatment
1	Cells were treated for 24 h followed by 1.0 mM of H_2_O_2_ exposure for 3 h
2	Cells were exposed concomitantly to each extract and 1.0 mM of H_2_O_2_ for 24 h
3	Cells were exposed to 1.0 mM of H_2_O_2_ for 3 h followed by cells treatment with each extract for 24 h. Evaluation of cell survival was performed using MTT assay as described above.

**Table 2:** Compounds present in extracts EtOH 0%, 50%, 80%, and 100%.

Name of Compound	Structure
Saturated fatty acids	
*n*-Hexadecanoic acid	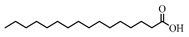
Octadecanoic acid	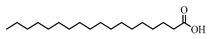
Polyunsaturated fatty acids	
9-Octadecenoic acid (E)-	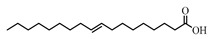
9,12-Octadecadienoic acid (Z,Z)-	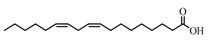
Esterified fatty acids	
9,12-Octadecadienoic acid, ethyl ester	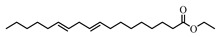
Hexadecanoic acid, methyl ester	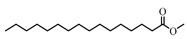
Hexadecanoic acid, ethyl ester	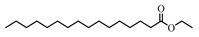
Phenolic compounds	
2-Propenoic acid, 3-(2-hydroxyphenyl)-, (E)-	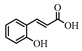
2-Methoxy-4-vinylphenol	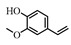
Ethanone, 1-(2-hydroxy-5-methylphenyl)-	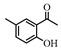
Volatile compounds	
Naphthalene, 3,4-dihydro-1,8-bis(trimethylsilyloxy)-	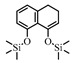
Benzofuran, 2,3-dihydro-	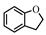
2,5-Dimethyl-4-hydroxy-3-(2*H*)-furanone	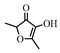
4*H*-pyran-4-one, 2,3-dihydro-3,5-dihydroxy-6-methyl	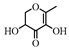
Sterols	
Stigmasta-5,22-dien-3-ol	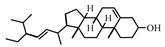
**Carbohidrates**	
3-*O*-Methyl-d-glucose	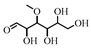
